# Comparison of 5‐year postoperative outcomes after Billroth I and Roux‐en‐Y reconstruction following distal gastrectomy for gastric cancer: Results from a multi‐institutional randomized controlled trial

**DOI:** 10.1002/ags3.12400

**Published:** 2020-09-15

**Authors:** Yutaka Kimura, Jota Mikami, Makoto Yamasaki, Motohiro Hirao, Hiroshi Imamura, Junya Fujita, Atsushi Takeno, Jin Matsuyama, Kentaro Kishi, Takafumi Hirao, Hiroki Fukunaga, Koichi Demura, Yukinori Kurokawa, Shuji Takiguchi, Hidetoshi Eguchi, Yuichiro Doki

**Affiliations:** ^1^ Department of Surgery Faculty of Medicine Kindai University Osaka‐Sayama Japan; ^2^ Department of Surgery Hyogo Prefectural Nishinomiya Hospital Nishinomiya Japan; ^3^ Department of Gastroenterological Surgery Osaka University Graduate School of Medicine Suita Japan; ^4^ Department of Surgery National Hospital Organization Osaka National Hospital Osaka Japan; ^5^ Department of Surgery Toyonaka Municipal Hospital Toyonaka Japan; ^6^ Department of Surgery Sakai City Medical Center Sakai Japan; ^7^ Department of Surgery Kansai Rosai Hospital Amagasaki Japan; ^8^ Department of Gastroenterological Surgery Higashiosaka City Medical Center Higashiosaka Japan; ^9^ Department of Surgery Osaka Police Hospital Osaka Japan; ^10^ Department of Surgery Minoh City Hospital Minoh Japan; ^11^ Department of Surgery Itami City Hospital Itami Japan; ^12^ Department of Surgery JCHO Osaka Hospital Osaka Japan; ^13^ Department of Gastroenterological Surgery Nagoya City University Graduate School of Medical Sciences Nagoya Japan

**Keywords:** Billroth I reconstruction, body weight, distal gastrectomy, gastric cancer, Roux‐en‐Y reconstruction

## Abstract

**Aim:**

We previously reported in a randomized controlled trial that Billroth I and Roux‐en‐Y reconstructions were generally equivalent regarding body weight change and nutritional status 1 year after distal gastrectomy for gastric cancer. We describe the long‐term follow‐up data 5 years after distal gastrectomy.

**Methods:**

We analyzed consecutive gastric cancer patients who were randomly assigned to undergo Billroth I or Roux‐en‐Y reconstruction after distal gastrectomy. We evaluated body weight change, nutritional status, late complications, quality of life (QOL) using the European Organization for Research and Treatment of Cancer Core QOL Questionnaire, and dysfunction using the Dysfunction After Upper Gastrointestinal Surgery for Cancer, 5 years after surgery.

**Results:**

A total of 228 patients (Billroth I = 105; Roux‐en‐Y = 123) were eligible for efficacy analyses in this study. Body weight loss 5 years after surgery did not differ significantly between the Billroth I and Roux‐en‐Y groups (10.0% ± 7.9% and 9.6% ± 8.4%, respectively; *P* = .70). There were no significant differences in other aspects of nutritional status between the two groups. Reflux esophagitis occurred in 19.0% of the patients in the Billroth I group vs 4.9% in the Roux‐en‐Y group (*P* = .002). Regarding QOL, Billroth I was significantly inferior to Roux‐en‐Y on the diarrhea scale (Billroth I: 28.6, Roux‐en‐Y: 16.0; *P* = .047). Regarding dysfunction, no score differed significantly between the two groups.

**Conclusions:**

Billroth I and Roux‐en‐Y reconstructions were generally equivalent regarding body weight change, nutritional status, and QOL 5 years after distal gastrectomy, although Roux‐en‐Y more effectively prevented reflux esophagitis and diarrhea.

## Graphical abstract

Billroth I and Roux‐en‐Y reconstructions were generally equivalent regarding body weight change, nutritional status, and quality of life 5 years after distal gastrectomy, excluding reflux esophagitis and diarrhea.

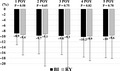

## 1 INTRODUCTION

Gastrectomy for gastric cancer is one of the most common gastroenterological operations in Japan.^1^ Among gastrectomy techniques, distal gastrectomy is most frequently performed, and Billroth I (BI) or Roux‐en‐Y (RY) are the main reconstruction methods after distal gastrectomy.^2^, ^3^ In BI reconstruction, food flows physiologically through the esophagus, stomach, and duodenum, but duodenal juice flows backward into the residual stomach because pyloric function is lost.^4^, ^5^ Conversely, in RY reconstruction, although there is no reflux of duodenal juice, Roux stasis syndrome is a possible complication.^6^, ^7^, ^8^ Each of these reconstruction methods has advantages and disadvantages, and the evaluation differs depending on the items being examined, such as body weight loss, nutritional status, gastritis in the remnant stomach, reflux esophagitis, and quality of life (QOL).^8^, ^9^, ^10^, ^11^, ^12^, ^13^ There is no clear answer to the question of which reconstruction method is better. Currently, the reconstruction method after distal gastrectomy is chosen according to the policies at each facility and the preference of the operator except in cases where the remnant stomach is very small or patients are at high risk.^3^

We previously reported finding no difference in weight loss, nutritional status, and QOL 1 year after surgery, but BI was often associated with reflux symptoms associated with gastritis in the remnant stomach and reflux esophagitis, as shown in randomized controlled trials evaluating BI and RY.^14^, ^15^ However, many studies comparing reconstruction methods were retrospective studies, and only a small number were randomized studies. To our knowledge, there have been no prospective long‐term comparisons of BI and RY with large samples. The aim of this study was to compare BI or RY as a reconstruction method after distal gastrectomy to evaluate long‐term changes in body weight and nutritional status, dysfunction, and QOL, 5 years after surgery.

## 2 MATERIALS AND METHODS

### 2.1 Subjects

We examined patients registered in a phase II randomized controlled trial (RCT) who underwent distal gastrectomy for gastric adenocarcinoma at the participating hospitals. This trial was a multi‐institutional RCT designed to compare the clinical effects of BI or RY reconstructive operations for gastric cancer resection.^14^, ^15^ The primary endpoint in the trial was postoperative body weight loss 1 year after surgery and the secondary endpoint was surgical morbidity. We also evaluated items related to nutritional status, such as serum albumin and lymphocyte count, as well as endoscopic examination findings of the remnant stomach and esophagus, postoperative QOL 1 year after surgery, and long‐term outcomes 5 years after surgery. Long‐term outcomes were set as a secondary endpoint in the protocol, which was amended during the case enrollment period. In the original study, we hypothesized that, compared with BI, RY may lead to 5% less body weight loss 1 year after surgery. The current study was conducted in consecutive patients recruited in the original trial. In this study, we provide the results of the final analysis of the 5‐year follow‐up data describing body weight loss, nutritional status, late complications, QOL, and dysfunction, 5 years after surgery.

Patients who required distal gastrectomy for gastric cancer with BI or RY reconstruction were eligible for this study. In other words, the eligibility criteria were that the tumor was localized in the middle or lower third of the stomach, with the expectation that a third of the stomach would remain after resection, and that the stomach could be reconstructed by either BI or RY. The outline of the trial is described below.^14^, ^15^ Patient eligibility criteria for the study were: (a) histologically proven primary adenocarcinoma of the stomach; (b) a lack of non‐curative surgical factors except for positive lavage cytology; (c) age between 20 and 90 years; (d) Eastern Cooperative Oncology Group performance status of 0‐1; (e) no prior chemotherapy or radiation therapy; (f) no history of gastrectomy or other malignancy (except carcinoma in situ of the uterus, cervical cancer, and focal adenomatous colorectal cancer) during the past 5 years; and (g) written informed consent.

Exclusion criteria were: (a) history of laparotomy (except appendectomy and laparoscopic cholecystectomy); (b) severe reflux esophagitis (Los Angeles [LA] classification grade A or higher); (c) interstitial pneumonia; (d) pulmonary fibrosis; (e) severe heart disease; (f) liver cirrhosis or active hepatitis; (g) chronic renal failure; and (h) severe diabetes (hemoglobin A1c [HbA1c]: ≥9.0%).

Patients were enrolled from 18 institutions belonging to the Osaka University Clinical Research Group for Gastroenterological Surgery. This study was conducted in accordance with the World Medical Association Declaration of Helsinki and was registered with clinical trial identification number UMIN000000878. The protocol of this study was approved by the institutional review board of each hospital. Written informed consent was obtained from all patients.

### 2.2 Randomization in the original trial

During surgery, surgeons confirmed that the eligibility criteria were met and that both reconstruction procedures could be chosen after distal gastrectomy, considering the length of the residual stomach before intraoperative randomization. After that, they immediately phoned the data center to receive a randomly generated assignment. Patients were intraoperatively randomized to either the BI group or the RY group. Randomization was performed using a minimization method according to body mass index and institutional preferences. Of the 332 patients enrolled from 18 hospitals, 163 were assigned to the BI group, and 169 were assigned to the RY group between May 2004 and October 2009.

### 2.3 Surgical procedure

In both groups, the surgeons performed standard distal gastrectomy with laparotomy or laparoscopic operations. Lymphadenectomy approaches were categorized as D1‐D3, as defined by the Japanese Classification of gastric carcinoma.^16^ The surgeons reconstructed by BI or RY according to the intraoperative allocation, and the reconstruction details were described previously.^14^, ^15^ Briefly, for BI reconstruction, the duodenum and remnant stomach were sutured, and for RY reconstruction, the jejunum was divided 20 cm distal to the ligament of Treitz, and gastrojejunostomy and jejunojejunostomy were performed. The oral portion of the jejunum was then anastomosed to the midjejunum, 30 cm distal to the gastrojejunostomy. There were no restrictions regarding an open or laparoscopic approach, hand‐sewn or stapling anastomosis, and antecolic or retrocolic routes during the RY reconstruction. In this study, all surgical procedures were performed or supervised by surgeons who were board certified by the Japanese Society of Gastroenterological Surgery and who were members of the Japanese Gastric Cancer Association. Laparoscopic surgery was performed or supervised by a qualified surgeon approved by the Endoscopic Surgical Skill Qualification system for clinical T1 early gastric cancer.

### 2.4 Follow‐up and data collection

Patients were followed for 5 years from the date of random assignment, and for as long as possible, thereafter. Adjuvant therapy was not specified in the protocol. Patients came to the hospital for examination at least once every 3 or 6 months for the first year after surgery. From the second year onward, patients were re‐evaluated at least every 6 or 12 months until 5 years postoperatively. Relapse was confirmed by imaging studies, including ultrasonography and/or computed tomography at least at 1‐year intervals until 5 years after surgery. Endoscopic examination was performed 1, 3, and 5 years after surgery to observe reflux esophagus (Los Angeles classification) and residual food in the remnant stomach.

We recorded patients' percentage body weight change from their pre‐surgical body weight to their weight 1, 2, 3, 4, and 5 years after surgery. Other nutritional status characteristics, such as serum albumin level, lymphocyte count, and prognostic nutritional index (PNI) were evaluated before and at 1 and 5 years after surgery. PNI was calculated as 10 × serum albumin level (g/dL) + 0.005 × lymphocyte count in peripheral blood (cells/mm^3^).^17^ Postoperative late complications ≥grade 2 according to the Common Terminology Criteria for Adverse Events version 3.0, recurrence, and survival were assessed from 1 to 5 years after surgery.^18^

### 2.5 Questionnaire survey

A self‐administered questionnaire that included the European Organization for Research and Treatment of Cancer (EORTC) Core Quality of Life Questionnaire (QLQ‐C30; Japanese version), which is a 30‐item cancer‐specific integrated system for assessing the health‐related QOL of cancer patients, and the Dysfunction After Upper Gastrointestinal Surgery for Cancer (DAUGS 20) questionnaire, which is a 20‐item questionnaire evaluating postoperative gastrointestinal dysfunction, was mailed to patients 5 years after registration. The details of the EORTC QLQ‐C30 and DAUGS 20 were described previously.^15^ In summary, the questionnaire for the EORTC QLQ‐C30 comprises five scales related to function (physical, role, cognitive, emotional, and social); three symptom scales (fatigue, pain, and nausea and vomiting); a global health and QOL scale; single items to assess additional symptoms commonly reported by cancer patients (dyspnea, appetite loss, sleep disturbance, constipation, and diarrhea); and perceived financial impact of the disease and treatment.^19^, ^20^ Higher scores indicated poorer QOL. The 20 items for the DAUGS 20 score were divided into the following seven categories: (a) diarrhea or soft feces, (b) pain, (c) dumping‐like symptoms, (d) food passage dysfunction, (e) nausea and vomiting, (f) decreased physical activity, and (g) reflux symptoms.^21^ Higher scores indicated more severe dysfunction.

A questionnaire survey was mailed from the clinical study registry center to patients who provided informed consent for the amended protocol. Patients completed the questionnaire and returned it by mail to the clinical study register center. To minimize bias, this questionnaire survey was not administered by a primary doctor.

### 2.6 Statistical analysis

All statistical analyses were performed with JMP Pro version 13.1 (SAS Institute Japan). Differences were considered significant at *P* < .05. Data were expressed as means ± standard deviation (SD). Fisher's exact test for categorical variables and the two‐sample *t* test for numerical variables were used to assess differences between the two groups, as appropriate. Total scores for the EORTC QLQ‐C30 and DAUGS 20 were compared between the two groups using the Mann–Whitney test.

## 3 RESULTS

### 3.1 Patients' characteristics

**FIGURE 1:**
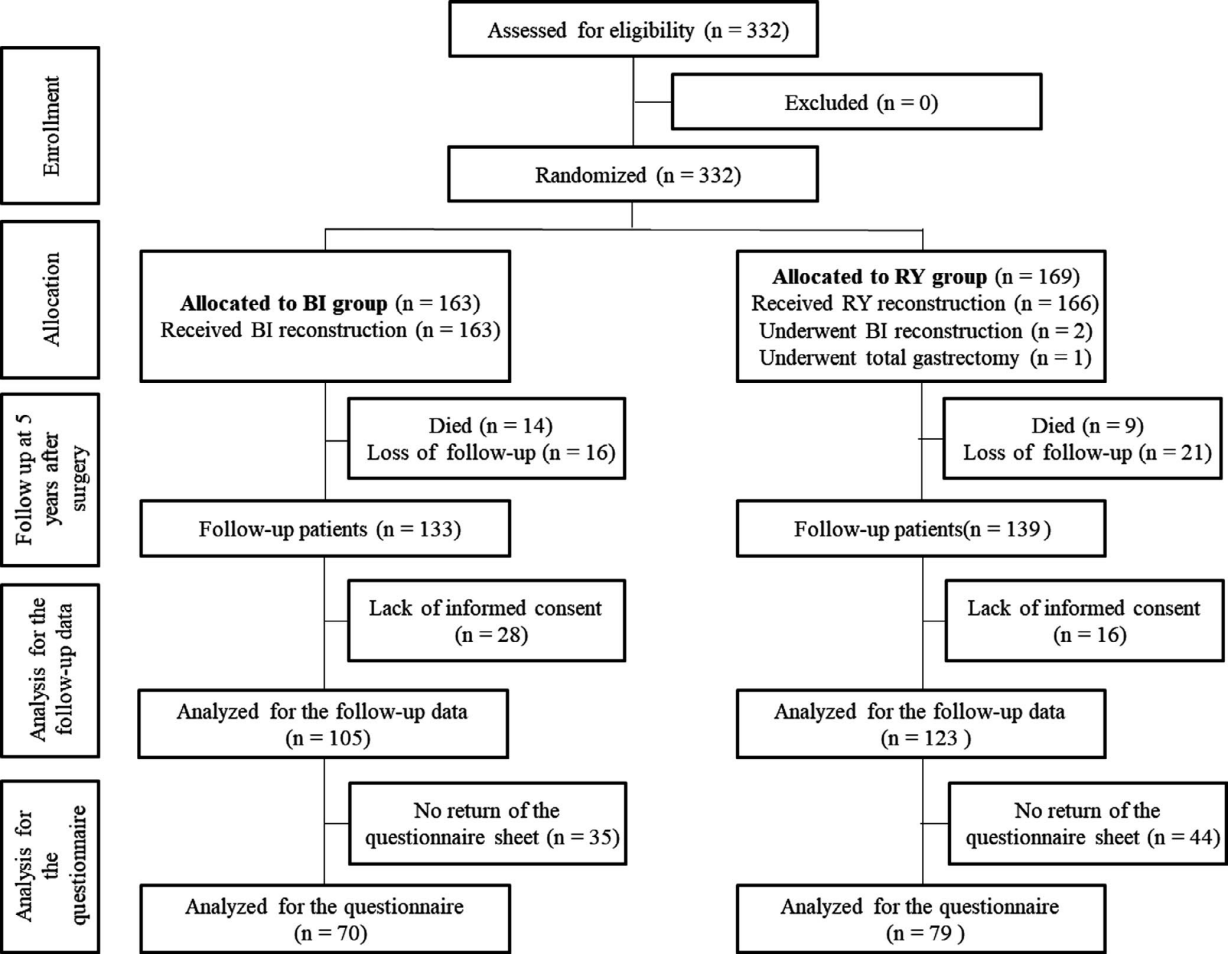
Study flow diagram. BI, Billroth I; RY, Roux‐en‐Y

A consort flowchart of the trial design is shown in Figure 1. Of the 332 patients who were registered in the original study, only 272 patients could be evaluated 5 years after surgery because of patients' death and loss to follow‐up. Nutritional indicators and body weight could be assessed in only 228 (83.8%) of the patients because of lack of informed consent for the amended protocol. The clinical features of the patients are shown in Table 1. There were 105 patients in the BI group and 123 patients in the RY group. The operation time was significantly longer in the RY group than in the BI group. Other characteristics were well‐balanced in both groups. R0 gastrectomy was performed in all cases.

**Table 1:** Patients' demographics and characteristics

	BI group (n = 105)	RY group (n = 123)	*P*
Age (y)	63.7 ± 8.5	63.7 ± 10.3	.98
Sex, male/female	72/33	80/43	.67
Preoperative serum albumin level (g/dL)	4.2 ± 0.4	4.2 ± 0.5	.53
Preoperative lymphocyte count (cells/mm^3^)	1943 ± 736	1941 ± 603	.98
Preoperative PNI	51.7 ± 6.0	51.0 ± 6.1	.63
Preoperative body weight (kg)	58.6 ± 9.8	59.3 ± 11.8	.64
Preoperative BMI (kg/m^2^)	22.4 ± 3.0	22.8 ± 3.2	.38
Tumor location, L/M	69/36	85/38	.69
Tumor size (cm)	2.9 ± 1.6	2.9 ± 1.6	.71
Lymph node dissection level, D1+/D2/D3	40/65/0	46/76/1	.65
Approach, open/laparoscopic	79/26	93/30	.99
Anastomosis, hand‐sewn/stapling	40/65	15/108	<.0001
Operation time (min)	179 ± 45	211 ± 45	<.0001
Operative bleeding (mL)	198 ± 242	209 ± 180	.7
pT, T1/T2/T3/T4a	80/16/4/5	90/13/13/7	.58
pN, N0/N(+)	86/19	96/27	.58
pStage, Stage I/ Stage II‐IV	91/14	102/21	.55
Postoperative adjuvant chemotherapy, yes/no	11/94	13/110	.99

Abbreviations: BI, Billroth I; BMI, body mass index; L, lower third; M, middle third; PNI, prognostic nutrition index; RY, Roux‐en‐Y.

### 3.2 Body weight change and nutritional status

**FIGURE 2:**
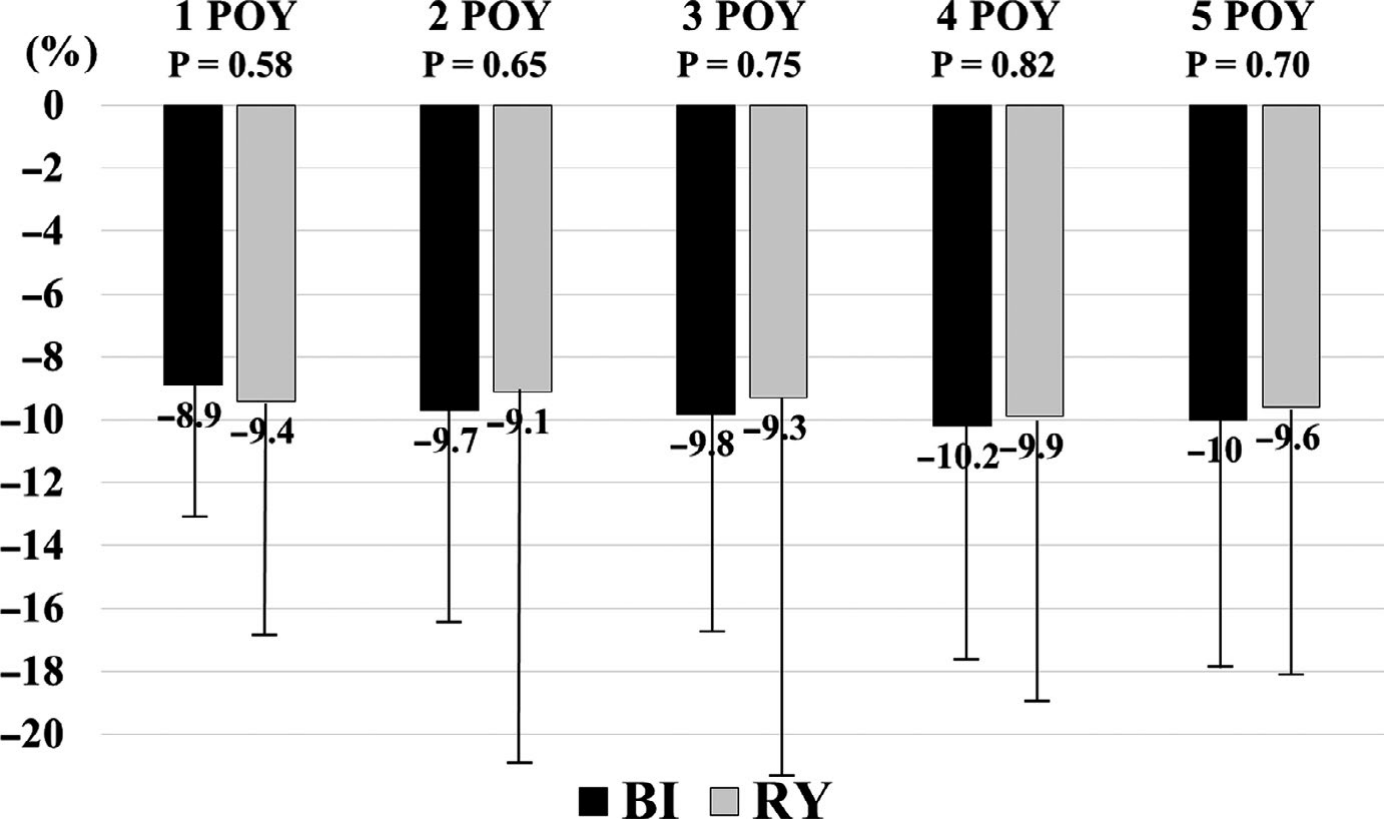
Percentage body weight change after surgery. BI, Billroth I; POY, postoperative year; RY, Roux‐en‐Y

The annual percentage body weight change for both groups is shown in Figure 2, from preoperative to the 5th year postoperatively. The percentage body weight change 5 years after surgery was −10.0% ± 7.9% for BI and −9.6% ± 8.4% for RY (*P* = .65), and there was no significant difference between the two groups at any point up to 5 years after surgery (Figure 2). Additionally, no significant body weight loss was observed in either group from the first year to the 5th year after surgery.

Serum albumin levels, lymphocyte counts, and PNI values did not differ after 5 years (Table 2), and there was no difference in nutritional status between 1 and 5 years after surgery in both groups.

**Table 2:** Postoperative nutrition status 5 y after surgery

		BI group (n = 105)	RY group (n = 123)	*P*
Serum albumin level (g/dL)	5 POY	4.2 ± 0.3	4.2 ± 0.3	.66
	Change	−0.05 ± 0.4	−0.01 ± 0.5	.49
Lymphocyte count (cells/mm^3^)	5 POY	1756 ± 626	1828 ± 540	.4
	Change	−148 ± 616	−102 ± 550	.58
Prognostic nutrition index	5 POY	50.5 ± 5.1	50.8 ± 4.6	.65
	Change	−1.0 ± 5.3	−0.7 ± 6.1	.68

Abbreviations: BI, Billroth I; POY, postoperative year; RY, Roux‐en‐Y.

There was no significant difference in long‐term weight loss or nutritional status according to the approach, anastomotic procedure, or route of reconstruction (data not shown).

### 3.3 Late complications

The incidence of late complications occurring by the 5th year after surgery did not differ overall, but BI was associated with more frequent reflux esophagitis compared with RY (Table 3).

**Table 3:** Postoperative late complications up to 5 y after surgery

	BI group (n = 105)	RY group (n = 123)	*P*
Total	24 (22.9%)	16 (13.0%)	.08
Dumping syndrome	2 (1.9%)	3 (2.4%)	1.00
Delayed gastric emptying	1 (1.0%)	2 (1.6%)	1.00
Ileus	2 (1.9%)	4 (3.3%)	0.83
Reflux esophagitis			
Endoscopic examination	20 (19.0%)	6 (4.9%)	.002
LA grade M/A/B/C/D	3/11/5/0/1	3/3/0/0/0	
Medication	5 (4.8%)	2 (1.6%)	.25
Pneumoniae	1 (1.0%)	1 (0.8%)	1.00
Cholecystitis	1 (1.0%)	2 (1.6%)	1.00
Others	1 (1.0%)	2 (1.6%)	.62

Abbreviations: BI, Billroth I; LA, Los Angeles classification; RY, Roux‐en‐Y.

### 3.4 QOL and gastrointestinal dysfunction

**FIGURE 3:**
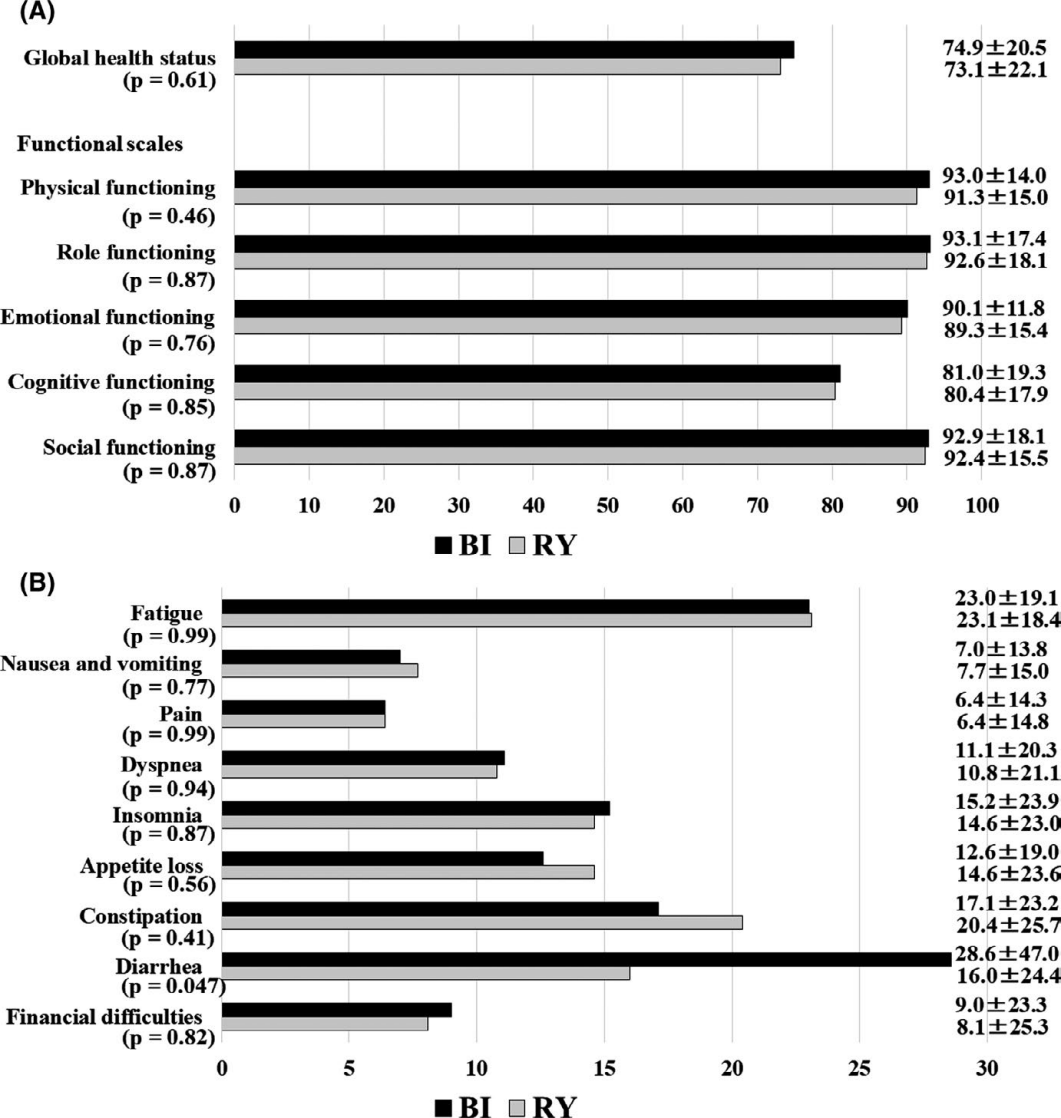
Postoperative quality of life scores evaluated using the European Organization for Research and Treatment of Cancer (EORTC) Core Quality of Life Questionnaire (QLQ‐C30) 5 y after surgery. A, Mean scores for global health status and functional scales. B, Symptom scales. BI, Billroth I; RY, Roux‐en‐Y

A questionnaire survey was administered to 105 patients in the BI group and 123 patients in the RY group who provided informed consent for the amended protocol and 149 patients (65.4%; 70 patients in the BI group and 79 patients in the RY group) responded to the survey. The mean scores for global health status were very similar in both groups 5 years after surgery (BI: 74.9 ± 20.5, RY: 73.1 ± 22.1; *P* = .61; Figure 3A). Regarding the functional scales, no significant difference was found in any of the five scales (physical, role, emotional, cognitive, and social functioning; Figure 3A). Regarding the symptom scales, BI was significantly inferior to RY on the diarrhea scale (BI: 28.6 ± 47.0, RY: 16.0 ± 24.4; *P* = .047; Figure 3B). There were no significant differences in the other eight symptom scales (fatigue, nausea and vomiting, pain, dyspnea, insomnia, appetite loss, constipation, and financial difficulties).

**FIGURE 4:**
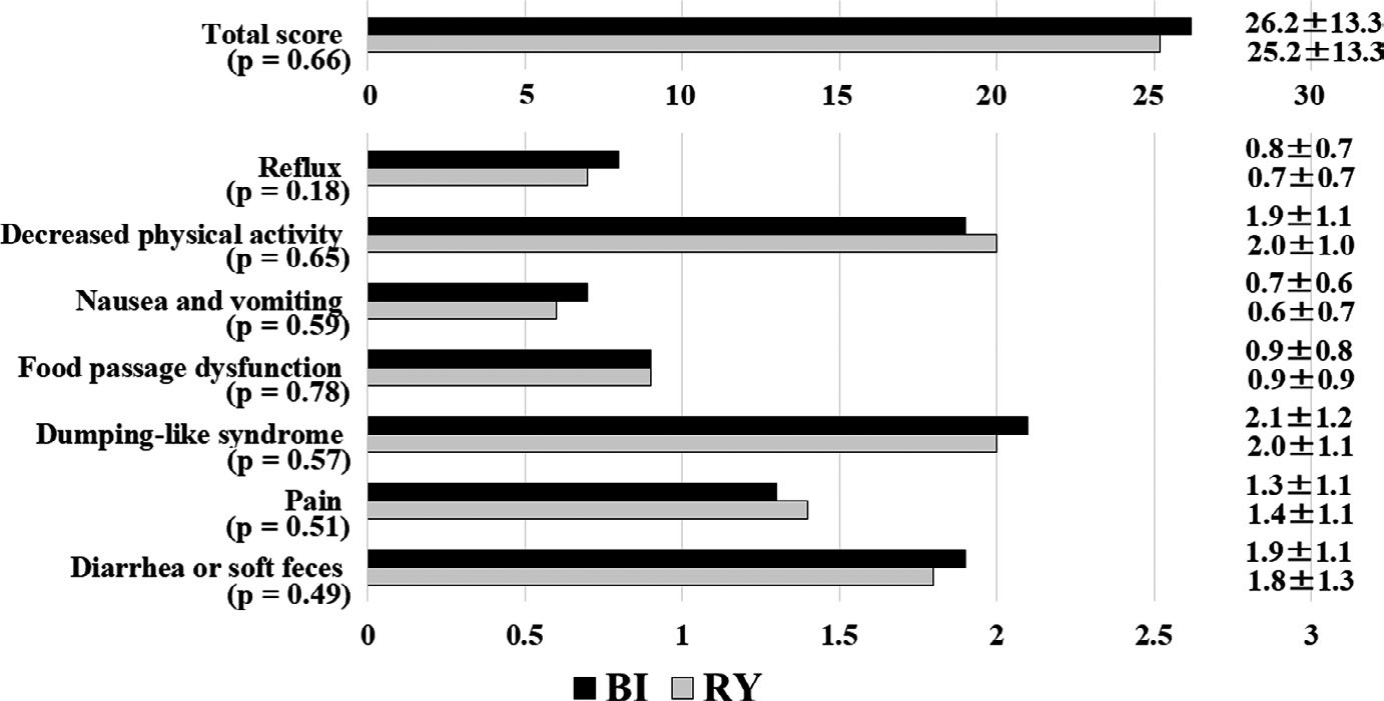
Postoperative dysfunction scores using the Dysfunction After Upper Gastrointestinal Surgery for Cancer (DAUGS 20) scoring system 5 y after surgery. BI, Billroth I; RY, Roux‐en‐Y

The total score for the DAUGS 20 was similar in both groups (BI: 26.2 ± 13.3, RY: 25.2 ± 13.3; *P* = .66; Figure 4). In the subclass analysis, there was no difference between the two groups for all items, 5 years after surgery.

There was no significant difference in QOL according to surgical techniques (data not shown).

## 4 DISCUSSION

This study, conducted as an adjunct to a multicenter randomized controlled trial evaluating the reconstruction method after distal gastrectomy, showed that there was no difference between BI and RY regarding long‐term weight loss and nutritional status. This finding was present even though RY was not superior to BI in terms of body weight change 1 year after surgery, as the primary endpoint in the original study. However, BI was inferior to RY regarding reflux esophagitis and diarrhea 5 years after surgery. We also found no difference in weight loss between the two groups from the first year to the 5th year after surgery, and there was no significant change in that time in either group. Several retrospective studies have compared BI and RY after distal gastrectomy.^10^, ^11^ This study provides valuable data because it involved a 5‐year long‐term follow‐up in a large number of prospectively randomized cases and because we investigated QOL in addition to long‐term postoperative weight loss and complications.

It is important to note that this study has limitations. First, we could not evaluate all patients because of death, loss to follow‐up, or lack of informed consent for the amended protocol. The transfer of several attending doctors who registered the case before the protocol revision was also considered a cause. Additionally, the questionnaire collection rate was low at 65.4%. Most patients who participated in this trial were followed in their respective hospitals, but it is possible that their interest in the trial diminished 5 years after surgery. Second, laparoscopic surgery is currently the standard surgical procedure for distal gastrectomy for gastric cancer, but at the time of case accumulation for this study, the proportion of patients undergoing laparoscopic surgery was low at less than 25%. Despite these limitations, our study should be useful to assist surgeons with deciding between the two procedures.

In the long‐term nutritional assessment, nutritional indicators such as serum albumin concentration did not differ between the two groups after 5 years as well as after the 1st year after surgery.^14^ There was also no difference in the weight loss rate between 1 and 5 years after surgery. It is generally known that body weight after gastrectomy decreases significantly in the early postoperative period and stabilizes after 6 months to 1 year; our results were consistent with previous reports.^10^, ^11^ A retrospective study reported that BI had a lower weight loss rate than RY, but it should be noted that there was bias in the operative choice in the study.^13^ Generally, RY is selected when the remnant stomach is small, and the size of the remnant stomach may affect the degree of weight loss. According to the results of an RCT by Nakamura et al, there was no significant difference in nutritional index 3 years after surgery, but weight loss following BI was significantly less than after RY.^9^ In that report, delayed gastric emptying was observed in 7% of the patients in the RY group, although there was no difference in early postoperative complications between the groups. In the patients with delayed gastric emptying, the prolonged fasting period may have led to greater weight loss in the early postgastrectomy period, which may have affected the difference in weight loss 3 years postoperatively in the RY group.

Regarding postoperative late complications, studies have reported no difference between BI and RY. Similarly, in our study, there was no difference between the two groups, including for ileus.^10^ In patients undergoing RY reconstruction, the tendency for ileus by hernia formation is thought to be enhanced because of mesenteric dissection, but in the current study, there was no difference in the occurrence of ileus. We believe this is because surgeons carefully closed the jejunojejunostomy mesenteric defect and the dorsum of the Roux limb (Petersen's space).

Regarding dumping syndrome, according to a questionnaire survey by Nunobe et al, more patients undergoing BI complained of symptoms compared with those who underwent RY for both early and late dumping, but there was no difference between the two groups in the survey of approximately 3000 cases using a postgastrectomy syndrome assessment scale (PGAS‐45).^12^, ^13^ In our study, the incidence of dumping syndrome did not differ between the two groups, with a rate of approximately 2%. Guidance to patients undergoing gastrectomy is important to prevent dumping syndrome, and the active implementation of nutritional guidance at each of our participating hospitals has led to a decreased incidence of this symptom. Additionally, the incidence of delayed gastric emptying seen in Roux stasis syndrome did not differ between the two groups and was shown to be less problematic long‐term, after surgery. Conversely, reflux esophagitis on endoscopy was more common 5 years after BI, as in previous reports.^11^, ^13^ However, few patients required medication for esophagitis, and there was no difference in reflux symptoms between the two groups in the gastrointestinal dysfunction questionnaire survey, using DAUGS 20; therefore, reflux may not be a clinically significant problem.

To evaluate QOL after gastrectomy, several objective scales, such as the EORTC QLQ‐C30, FACT, and PGSAS‐45 questionnaires, have been developed. In this study, we used the EORTC QLQ‐C30, which has high power and has been used in clinical trials.^22^, ^23^, ^24^ Many clinical studies showed no significant difference between the BI and RY groups except for reflux, but few studies evaluated QOL 5 years after surgery. In an RCT by Nakamura et al, according to the FACT questionnaire results, patients who underwent RY had a worse QOL score for diarrhea 3 years after surgery, but there was no difference regarding diarrhea between the two groups in the evaluation using the PGAS45.^9^, ^13^ Conversely, in this study, only the QOL score for diarrhea was worse in the BI group compared with the RY group. The SD value for the QOL score in the BI group was very large, and we considered that this was caused by including some patients in the BI group who felt that their QOL was significantly decreased regarding diarrhea.

Although there are limitations, this study provides useful information regarding reconstruction methods and long‐term nutritional status, postoperative disability, and QOL after surgery. In summary, BI and RY after distal gastrectomy for gastric cancer were generally equivalent regarding postoperative body weight loss and nutritional status 5 years after surgery, although RY more effectively prevented reflux esophagitis and diarrhea.

## DISCLOSURES

Funding: The authors declare that this study was not funded.

Conflicts of Interest: Authors declare no conflict of interests for this article.

Author Contribution: Conceived and designed the study: Y. Kimura, S. Takiguchi, and Y. Doki. Provision of study materials and patients: Y. Kimura, M. Hirao, H. Imamura, J. Fujita, J. Matsuyama, K. Kishi, T Hirao, H Fukunaga, K Demura, and M. Yamasaki. Assembly of data and critical revision of the article: H. Eguchi and Y. Doki. Statistical analysis of the data: J. Mikami and Y. Kurokawa. Statistical analysis and interpretation of the data: Y. Kimura, M. Yamasaki, and S. Takiguchi. Final approval of the manuscript version to be published: Y. Kimura. All authors approved the final version of the manuscript.

Approval of the Protocol: The protocol for this research project has been approved by a suitably constituted Ethics Committee of the institution and it conforms to the provisions of the Declaration of Helsinki.

Informed consent: Informed consent was obtained from all the subjects.
